# Decoding the influence of emotional and attentional states on self-control using facial analysis

**DOI:** 10.1038/s41598-024-73729-6

**Published:** 2024-10-26

**Authors:** Gökhan Aydogan, Janek Kretschmer, Gene Brewer, Samuel M. McClure

**Affiliations:** 1 Department of Economics, Zurich Center for Neuroeconomics, University of Zurich, Zurich, Switzerland; 2 Department of Finance, School of Business and Economics, Maastricht University, Maastricht, The Netherlands; 3 Department of Psychology, Arizona State University, 950 S. McAllister Ave, Tempe, AZ 85287 USA; 4 Department of Psychology, University of California Riverside, Riverside, United States

**Keywords:** Self-control, Attention, Fatigue, Ultimatum game, Anagram task, Psychomotor vigilance task, Emotion, Motivation, Social behaviour, Human behaviour, Attention, Cognitive control

## Abstract

Self-control plays a pivotal role in pursuing long-term goals related to health and financial well-being. While ample evidence suggests that humans are prone to occasional self-control lapses, little is known about how changes in emotional and attentional states affect the ability to maintain self-control. In two studies (*N*_*1*_ = 109 and *N*_*2*_ = 90), we used emotion recognition software to decode participants’ facial expressions while manipulating their attentional and emotional states during a Psychomotor Vigilance Task (PVT) before exerting self-control in a subsequent task. Our findings reveal dissociable roles of attention and valence in maintaining self-control, depending on the distinct demands of the task. Specifically, performance in a subsequent cognitive task was predominantly associated with changes in attentional states during the PVT rather than valence. Conversely, preferences in a subsequent social task were associated with changes in valence states during the PVT, while attention showed no effect. This dissociation between attention-invoked and emotion-invoked lapses in self-control holds significant implications for psychological and economic models of self-control, ultimately contributing to the development of policies to mitigate the detrimental consequences of individual self-control lapses.

**Supplementary Information:**

The online version contains supplementary material available at 10.1038/s41598-024-73729-6.

## Introduction

Self-control lapses have detrimental effects on health, maintaining positive relationships, achieving educational goals, and financial well-being^1,2^. For instance, virtually omnipresent food temptations in modern societies are considered to have substantially contributed to the epidemic of obesity^3,4^, with deleterious health and economic consequences amounting to more than $147 billion annually in the US alone^5^. Similarly, poor financial decision-making is a main source of insufficient retirement savings, irrespective of the total available lifetime income^6^. Moreover, substance abuse and addiction - a state characterized by substantial loss of control - is estimated to impose an economic burden of more than $740 billion annually in the US^7,8^. It is suggested that the common underlying factor leading to these poor outcomes constitutes insufficient self-control, which serves as an umbrella construct that subsumes concepts from different disciplines^9,10^. Here, we refer to the common notion of self-control as the capacity for regulating behavioral, attentional and emotional impulses (of the self by the self) to achieve long-term goals^10^.

Although previous evidence points to neuroanatomic^11,12^ and genetic^13^ roots of self-control, little is known as to what directly triggers self-control lapses, and whether there are distinct underlying mechanisms that differentially affect those lapses. Specifically, it is still not well understood how momentarily changes in affective or attentional states would trigger self-control lapses^14,15^. Therefore, a primary goal of the current work was to assess dynamic change in emotional and attentional measurements via software that decodes these components from a recording of participants’ faces while they completed a sequential task paradigm that taxes executive control in the first phase and examines transfer to performance in a new task in the second phase.

Here, we aim to address this question by manipulating and measuring dynamic changes in emotional and attentional states of participants while they were exerting self-control in a cognitive as well as in a social task^10^. We specifically build upon the sequential task paradigm^16^. In this paradigm, participants complete a vigilance task where changes in performance can be unambiguously assessed (i.e., depletion is reflected by performance deterioration across the task) and the degree of deterioration in performance in this initial vigilance task is associated with performance in a subsequent vigilance task (i.e., negative transfer). Thus, the standard paradigm can be used to assess how exerting effort in executive control during an initial task can diminish attention and lead to goal neglect, resulting in lapses of self-control in a subsequent task^2,17^.

However, in our study, we depart from conventional approaches using a standardized Psychomotor Vigilance Task (PVT) by experimentally manipulating participants’ motivation through *sham feedback* on their task performance. That is, unlike previous studies on depletion effects using a standard PVT^16^, we followed a different approach by incorporating a motivational manipulation in the form of sham feedback to influence performance and attentional and emotional states during the task^18^. This provides a type of experimental validation of our measures collected during the initial task and allows us to examine their influence on subsequent tasks. Critically, this modified initial task design does not aim to influence depletion effects but rather serves as a means to influence participants’ affective and attentional states through the provision of sham feedback on their initial task performance.

**Fig. 1:**
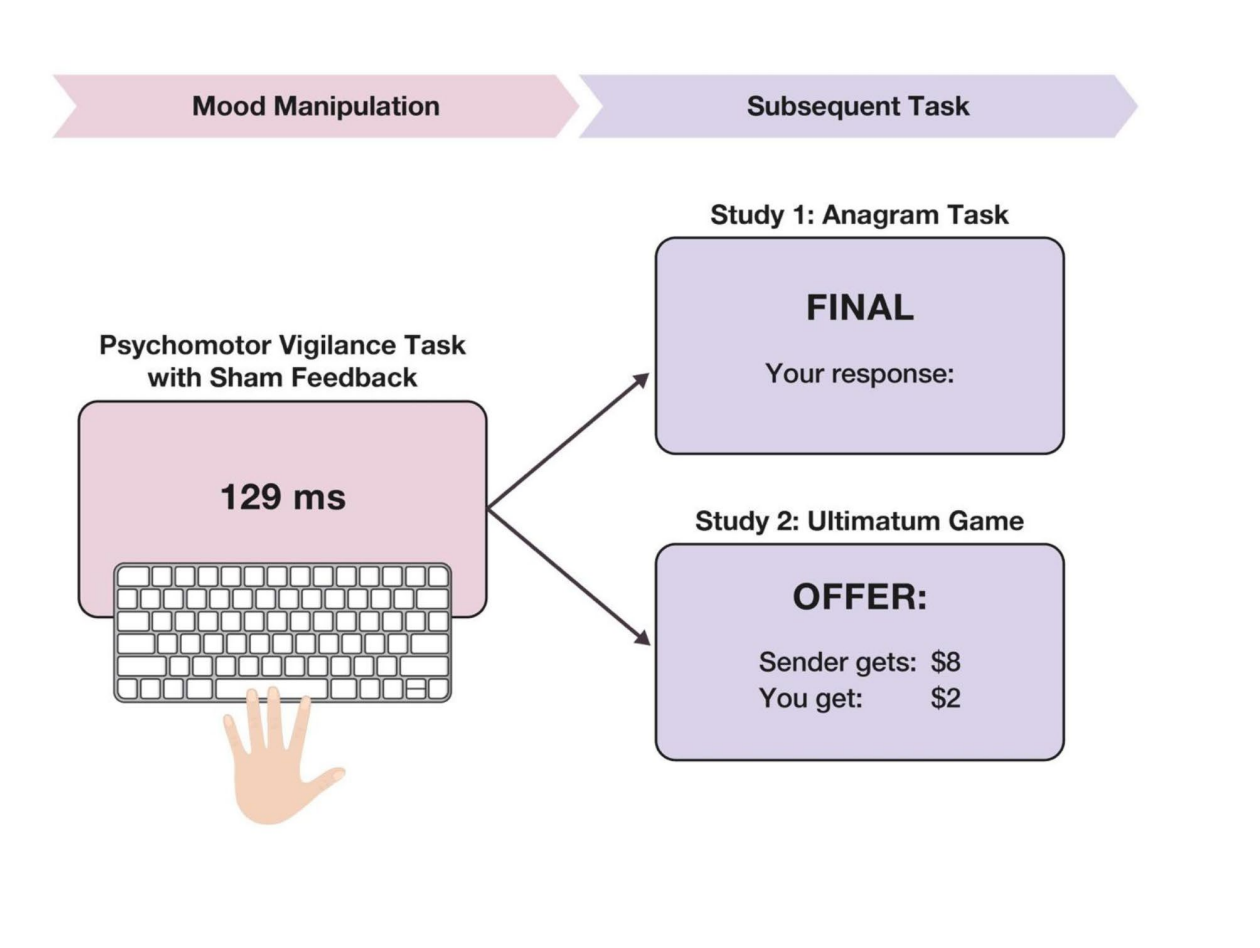
The sequential task paradigm: Both studies, 1 and 2, consisted of two parts. In the first part (i.e., initial task), we asked participants to perform the Psychomotor Vigilance Task to induce mental fatigue through continued engagement of motivational and executive functions. During this task, we manipulated participants’ emotional and attentional states by randomly assigning either a positive or negative (sham) feedback treatment. In the second part (i.e., subsequent task), participants were asked to either perform the Anagram Task (Study 1, *N* = 109) or the Ultimatum Game (Study 2, *N* = 90).

Figure 1 shows the general procedure in the sequential task paradigm, which consists of two parts. In the first part of the experiment, we employed the modified PVT with (sham) feedback to manipulate the emotional and attentional states of the participants. To achieve this, the participants were randomly assigned to receive either positive or negative (sham) feedback while they were performing the PVT (see details in Methods). Previous research suggests that positive and negative feedback have direct effects on emotional states and subsequent task engagement^19–21^, which we leveraged to induce an exogenous shift in participants’ attention and valence with the use of (sham) feedback during PVT.

In the second part, participants were asked to perform either the Anagram Task (Study 1, *N* = 109) or the Ultimatum Game (Study 2, *N* = 90) to measure different aspects of self-control in a subsequent task. This sample size is comparable to or even above similar studies examining the link between emotions and behavior in the laboratory^22–24^. We employ the Anagram Task as a self-regulatory task because it reflects the capacity to persist in effortful control when facing *cognitive difficulty*, which is a core aspect of self-control^10,25^. Therefore, we hypothesize that task-induced changes in attention and valence would affect maintaining self-control in an effortful task, and therefore would reduce observed performance in the Anagram Task.

Similarly, self-control plays a pivotal role in the Ultimatum Game (UG). Specifically, the receiver in the UG is challenged to regulate immediate impulses when faced with unfair offers. In line with this notion, previous fMRI experiments showed that rejections of lower offers are associated with increased activity in brain areas typically involved in (negative) emotion processing^26–29^. Other studies analyzing skin conductance (a measure of emotional arousal) also showed higher emotional arousal when subjects encountered unfair offers with ensuing higher rejection rates^30,31^. Hence, it can be argued that receivers need to *resist* their immediate impulse to punish unfair co-players (i.e., short-term temptations) if they want to maximize their (long-term) monetary payoffs. Thus, self-control seems to be crucial to regulate emotional impulses when monetary long-term benefits are at stake^32–34^. Therefore, we hypothesize that changes in attention or valence induced by the feedback treatment in the PVT affect self-control, which in turn would influence behavior in the subsequent tasks (Anagram Task or UG). Specifically, this should materialize as higher rejection rates and fewer correctly solved anagrams due to lower self-control.

### Part one - the initial task

 We used the standard PVT in which we attempted to experimentally manipulate participants’ emotional and attentional states using sham feedback regarding their performance. Specifically, we gave participants either negative or positive (sham) feedback while they were performing the PVT^18,35^. In the PVT, participants were instructed to monitor a computerized stopwatch that begins counting at random intervals in milliseconds and to stop the counter as soon as it starts by pressing a key on the keyboard^35–37^. In contrast to this standard version of the task, we told participants that they would have to perform this task for a certain time period but could finish the task earlier (positive feedback) or later (negative feedback) when they reacted faster or slower than a displayed time threshold^14^. We employed an adaptive calibration method to establish a time threshold that would primarily lead to success (positive feedback) or failure (negative feedback) in this task while ensuring a fixed task duration of 20 min for all participants. That is, the actual elapsed time for the task was held constant across both treatment conditions (refer to Supplementary Material S2), with the only difference being the performance feedback provided in terms of the prospects for task completion time. Participants in the negative feedback treatment were informed that they had to perform the task for 5 min but mostly received sham responses about their performance. This resulted in a time penalty of an additional 15 min. Conversely, participants in the positive feedback treatment were informed that they had to perform the PVT for 35 min, but they mostly received positive feedback, resulting in a time reduction of 15 min. Thus, the actual elapsed time for the task was precisely 20 min for both groups. We opted not to incentivize the PVT for two reasons: (i) we wanted to ensure that participants started the second subsequent task on an even footing, without any differences in their earning levels and (ii) we aimed to manipulate their emotional and attentional states without interference from financial considerations (e.g., regret).

During the PVT, we measured the attentional and emotional states of participants with emotion recognition software, which enabled us to collect objective indicators for attentional and emotional states with a relatively high sampling rate of 24 frames per second. Thus, this approach enabled us to detect extremely short and subtle affective expressions in the human face (e.g., micro expressions). To analyze the video data, we used the emotion recognition software library *Affectiva -* a tool that provides values for valence, all seven basic emotions, and attention toward the visual display^38,39^. The measure of the attentional state is derived from the head angle and orientation relative to the screen. Besides attention, we restricted our analysis to valence - a generic indication of positive or negative affect to assess emotional states.

### Part two - the subsequent task

 To examine the effect of the treatment in part 1, we instructed participants in the second part of the study to perform either an Anagram Task (Study 1, *N* = 109) or play an incentivized UG (Study 2, *N* = 90, with 45 in either role. For more details, see Methods). That is, we examined (a) near transfer effects from the PVT to a related cognitive task (i.e., the Anagram Task) and (b) investigated far transfer from the PVT to an incentivized social decision-making task (i.e., UG). This would allow us to show that lapses in self-control might, in part, depend on an *objectively* measurable level of attention or valence^1,25^. To go beyond pure correlational inference, we additionally manipulated participants’ attentional and affective states by providing them either with positive or negative feedback during the PVT.

## Results

### Testing the effectiveness of sham feedback on attention and valence during the PVT

**Fig. 2:**
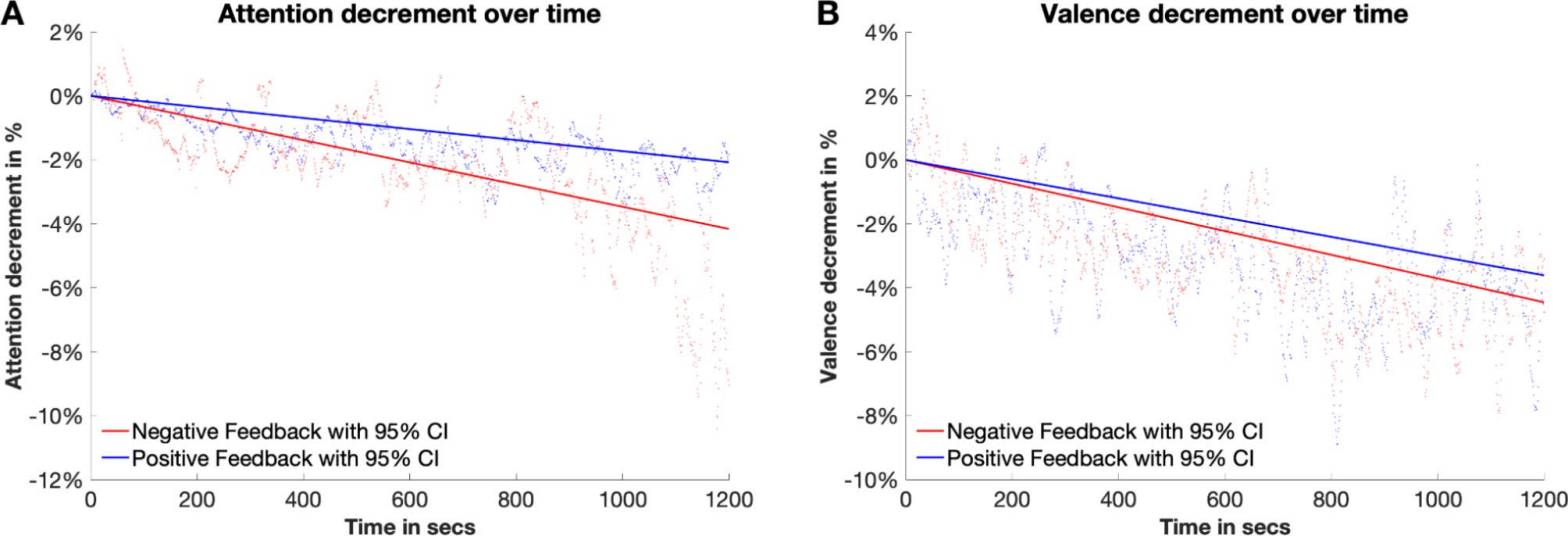
The figure shows the decline in attention (Panel A) and valence (Panel B) among 211 participants (combined from Studies 1 and 2) during the Psychomotor Vigilance Task. This significant decline is highlighted by the fitted regression lines (solid lines). The dotted lines represent the average attention and valence levels over time, aggregated across all participants. Both measures, objectively assessed using emotion recognition software, demonstrate a notable decrease, with a more pronounced decline observed in the negative (sham) feedback condition compared to the positive (sham) feedback condition.

First, we examined the effectiveness of the treatment for both studies 1 and 2 and tested whether the manipulated (sham) feedback would modulate *attention* or *valence* while participants performed the PVT. Specifically, using emotion recognition software, we elicited shifts in these attentional and emotional states during the PVT of all 211 participants across both studies. Further, based on these objective measures, we computed for each participant separately their shift in attention and valence, normalized for their respective baseline levels. Figure 2 illustrates the temporal evolution of attention and valence in percentage changes (normalized at baseline) throughout the PVT, revealing a consistent decrease in both measures. A fixed effects regression with random intercepts confirms a significant decline in attention (*β*_*Time*_ = − 0.004, *p* < .001) and in valence (*β*_*Trial*_ = − 0.0037, *p* < .001) over time during the PVT. Further, our results indicate a stronger decrement in valence and attention when participants were given negative (sham) feedback as opposed to positive feedback, confirming a successful manipulation in affective and attentional states of participants (for attention: *β*_*treatment*_ = 0.003, *p* < .001; for valence: *β*_*treatment*_ = 0.0005, *p* < .001).

### Study 1: performance in the Anagram Task after the PVT

We then turned to Study 1 and tested whether participants’ *attention* or *valence* during the PVT would affect their performance in the Anagram Task. Using a multilevel mixed-effects linear regression model, we predicted performance in the Anagram Task based on treatment, and their respective attention and valence recordings during the PVT (see Models 1 & 2 in Table 1).

The regression analysis in Table 1 (Model 1) reveals that there was no treatment effect on Anagram Task performance (*p* > .1). However, attention during the PVT (Model 2) was significantly associated with performance in the Anagram Task (*p* < .01), while valence exhibited no significant influence on performance in the task (*ps* > 0.1). Thus, a decrease of attention during the PVT lowered performance in the subsequent Anagram Task, indicating that participants who were able to maintain attention control throughout the PVT were also more likely to perform better in the Anagram Task.

**Table 1:** Predicting anagram performance.

Predictor	Dependent Variable: Correctly solved anagram puzzles	
	Model (1)	Model (2)
Treatment condition (1 = pos. or 0 = neg. feedback)	0.645 (1.458)	0.437 (1.460)
**Attention during PVT**		0.110** **(0.039)**
Valence during PVT		0.0148 (0.022)
Constant	19.272** (1.002)	9.053* (3.563)
Log Pseudolikelihood	-34,288	-34,233

Note: The table reports unstandardized regression coefficients, with clustered standard errors on subject level in parentheses (*N*_*Participants*_ = 109). *p < .05. **p < .01.

Further, our results indicate that valence during the PVT had no effect on performance in the Anagram Task (*p* > .1), indicating that shifts in mood had no impact on performance in the subsequent cognitively demanding Anagram Task.

**Fig. 3:**
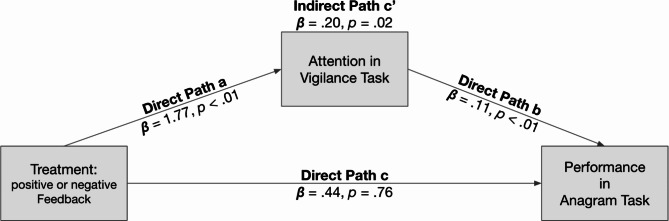
Mediation analysis of attention mediating the effect of treatment on performance in the Anagram Task (path c’). Analysis was conducted on all trials with standard errors clustered on subject level (*N*_*Obs*_*=* 9,955; *N*_*Clusters*_ = 109). All values were calculated using the Stata 18.0 command SEM.

Using structural equation modeling, we then tested whether the attention also mediated the effect of feedback (positive or negative) on performance in the Anagram Task (see Fig. 3), since attention was associated with both, the treatment (path a) and performance in the subsequent Anagram Task (path b). A mediation analysis revealed an indirect effect of treatment on performance in the Anagram Task (*p* = .02), although no direct association was present (path c) between both variables (*p* = .76). Although rather counterintuitive, recent methodological research has postulated and described significant mediation in the absence of a total effect^40–42^. In sum, this data suggests that near task transfer (i.e., performance across cognitive tasks) is affected by attention, and not by valence.

### Study 2: preferences in the Ultimatum Game after the PVT

In Study 2, we aimed to investigate the effect of attention and valence during the PVT on social preferences in an UG (*N* = 90). As in Study 1, we randomly assigned participants to either a positive or negative feedback treatment during the PVT. Afterwards, we asked participants to play two rounds of an UG, either as a sender or as a responder (see Methods of Study 2 for further details). We limited our analysis to the behavior of responders since previous literature indicates that altruistic punishment (i.e., punishment of unfair behavior) is closely related to emotional reactions to perceived unfairness^34,43^.

To test the effect of attention and valence states on behavior in the subsequent UG, we ran a Random-effects probit regression (see Methods of Study 2 for further details) with the responder’s decision to accept as a dependent variable, and with treatment (pos. or neg. feedback), valence and attention during the PVT as independent variables, while controlling for the size of an offer (see Models 1–3 in Table 2). The regression analysis in Table 2 (Model 1) reveals that there was no significant treatment effect (*p* > .1). Further, we found that attention did not influence the inclination to accept an offer. Then, we regressed acceptance rates on valence (Model 2 in Table 2), and found that recorded valence during the PVT predicted acceptance rates in the UG, indicating that participants who show a larger negative emotional response to the PVT are also less likely to accept a (lower) offer (*p* < .01).

**Table 2:** Predicting a responder’s inclination to accept an offer.

Predictor	Dependent Variable: Acceptance of offer	
	Model (1)	Model (2)
**Offer size**	1.230** **(0.406)**	1.235** **(0.397)**
Treatment condition (1 = pos. or 0 = neg. feedback)	0.231 (0.553)	0.537 (0.469)
Attention during PVT		0.112 (0.073)
**Valence during PVT**		0.158* **(0.063)**
Constant	-2.921** (0.958)	-13.108 (7.367)
Log Pseudolikelihood	-59.15	-49.34

Note: The table reports unstandardized coefficients of Random-effects probit regression, with robust standard errors clustered on participant level in parentheses (with *N* = 45 participants in the role of responders). *p < .05. **p < .01.

**Fig. 4:**
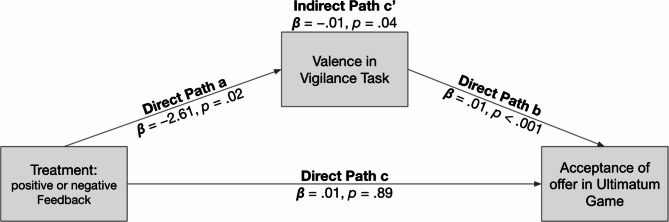
Mediation analysis reveals that *valence* significantly mediates the effect of treatment on the inclination to accept an offer (path c’). Analysis was conducted on all trials with standard errors clustered on subject level (*N*_*Obs*_ = 32,888; *N*_*Clusters*_ = 45). All values were calculated using the Stata 18.0 command SEM.

We further investigated whether valence mediated the relationship between treatment and the likelihood of accepting an offer in the UG. The results showed that valence indeed mediated the effect of treatment on the acceptance rate (indirect path c’, *p* = .04). Consistent with Study 1, we found no direct effect of (sham) feedback on respondents’ behavior in the UG, as depicted in Fig. 4.

## Discussion

Self-control is a vital element in achieving long-term goals related to health, financial well-being, and positive relationships^11–13^. However, lapses in self-control are prevalent, and it remains unclear how alterations in attentional and emotional states affect one’s ability to maintain self-control^1,2,16,44^. To shed light on this, we conducted a study manipulating participants’ attentional and emotional states before they exerted self-control in a cognitive and social task^45^. Our findings revealed that attention and valence play dissociable roles in maintaining self-control, depending on the task demands. Specifically, our results indicate that (a) the feedback manipulation had a negative effect on participants’ attention and valence^19–21^, and (b) that the PVT induced attention and valence levels altered participants’ behavior in a subsequent cognitive task (near transfer) as well as in a social task (far transfer).

Firstly, we observed a significant shift in participants’ attention and valence during the PVT across both experiments, suggesting a potential decline in intrinsic motivation^1,16,25^. Specifically, we argue that participants may initially be driven by the novelty and interest of the task (a “want-to” goal). However, as the task is perceived more monotonous over time, this intrinsic motivation diminishes, causing a shift in focus that reclassifies the task as a “have-to” goal, ultimately leading to poorer performance. This phenomenon has previously been associated with increased reaction times during the PVT. Our study extends this understanding by demonstrating a significant decline in objective measures of valence and attention, as indicated by micro facial expressions.

Secondly, in contrast to previous research that investigated how self-control lapses manifest in emotional or attentional states^25,46–50^, our study suggests that negative emotional or attentional states constitute a potential driving factor underlying self-control lapses. Using emotion recognition software (Affectiva), we objectively detected these shifts and found that attentional states affect performance in a subsequent cognitive task (i.e., the Anagram Task), while emotional states predominantly affect performance in a social task (i.e., the UG). Furthermore, this method allowed us to unobtrusively elicit those variables while avoiding experimenter demand effects or measurement inaccuracies that could stem from self-reports^1^.

Thirdly, in both studies, we identified a significant mediation effect in the absence of a direct effect of the feedback treatment on subsequent behavior. Specifically, in both studies, the objectively derived measure for valence (respectively attention) mediated the effect of the feedback treatment on performance in the subsequent task. Additionally, we might have detected a possible suppression mediation effect in Study 2, although the direct path was not significant^51^. Suppression mediation effects typically manifest when the direct and indirect effects display opposite signs. In Study 2, we observed that the potential suppressor, i.e., participants’ valence levels, increased the treatment manipulation’s predictive capacity concerning subsequent task performance. In this case, the mediator might have, therefore, suppressed the total effect of the manipulation on the subsequent task performance. Further, a positive mediation effect in the absence of a direct (or total) effect highlights the importance of examining all possible underlying mechanisms, including suppression mediation, in self-control studies. Ignoring potentially mediating factors such as attention or emotions could leave important underlying mechanisms undetected^41,42^. While we do not claim that this finding is the general case for all self-control studies, it emphasizes the need for a thorough investigation of all potential underlying mechanisms.

The mediation analysis revealed that there are no direct but only indirect effects of the treatment (positive or negative feedback in the PVT) on task performance. However, the mediation models also highlight that the sham feedback manipulation indeed affected facial measures of attention and valence. Notably, participants who exhibited greater changes in these facial measures tended to show a more pronounced negative transfer to task performance in both the Anagram Task and the UG. Thus, by placing subjects in challenging situations demanding self-control, we were able to investigate the repercussions of self-control lapses on behavior in both social (e.g., the UG) and cognitive contexts (e.g., the Anagram Task). The considerable variation in individual responses to the treatment may explain the absence of a direct effect and suggests that not every participant may react the same way to the exogenous (sham) feedback. This finding emphasizes the need for eliciting direct responses (such as emotional or attentional states) to treatments when studying self-control lapses. In other words, to shed light on the causes and consequences of self-control lapses it is paramount to directly (and objectively) measure participants’ reaction (and efficacy) to those treatments.

However, limitations of this study include the inability to exclude alternative explanations for the observed treatment effects. Albeit any shifts were causally induced by the treatment, it is possible that the treatments themselves elicited, for example, a sense of disbelief among participants regarding the fairness of the environment (or of the experiment itself) due to the deception used in the experiments. This may have in turn influenced rejection rates in the UG. However, facial expressions collected during the PVT demonstrate that indeed the treatment induced shifts in valence and attention, and therefore, it seems plausible that these shifts were directly related to behavior in the subsequent tasks.

Finally, our results support the hypothesis that self-control is not a uni-dimensional, but rather a multi-faceted phenomenon that consists of at least attentional and affective components. Based on our findings, we argue that a more process-oriented approach that utilizes physical (non-behavioral) data would solve, or at least mitigate, this issue. Moreover, our study also has important practical implications. Our findings suggest that strategies for improving self-control need to consider task-specific demands. For instance, interventions that aim to improve cognitive self-control may need to focus on attentional training, whereas interventions that aim to improve social self-control may need to focus on emotional regulation^48,52–55^. In conclusion, we think that these findings may have significant implications for psychological and economic models of self-control and may inform the development of more effective strategies for improving self-control in different contexts.

## Methods

### PVT: extraction of valence and attention using facial cues

To objectively quantify measures of valence and attention during a sustained attention task, we conducted an analysis of video footage recorded while participants performed the PVT. The PVT is a simple reaction time task that imposes minimal demands on the cognitive system^56^. During this task, participants were instructed to monitor a computerized stopwatch that begins counting at random intervals in milliseconds and to stop the counter as soon as it begins counting by pressing the spacebar. Previous studies have reliably shown that participants’ performance in the PVT decreases over time, thereby validating its effectiveness as a task for inducing decrement in participants’ attention^35–37^.

We use Affectiva, a software that integrates various biosensors such as facial expressions (e.g., smile and brow furrow), head angle, and eye tracking to generate numerical scores of participants’ valence and attention from their video data. This approach captures emotional, physiological, and cognitive responses in real time as stimuli are presented. Thus, by employing Affectiva, we can conduct precise, time-locked analyses linking participants’ reactions, emotional states, and attention levels with specific events during the experiment (for more details, see Methods).

Affectiva’s deep learning algorithms have undergone extensive training with diverse data sourced from a global dataset of over 14 million videos collected across 90 countries (Affectiva’s specific algorithms are proprietary information. For more details see: https://www.affectiva.com/product/qualitative-research*).* Affectiva’s ability to accurately classify emotions, especially anger and happiness, has been validated against results obtained from facial electromyography^57,58^. It, therefore, has been used in previous clinical studies to examine individuals’ facial expressions during various social^59^, and cognitive tasks^60,61^.

**Fig. 5:**
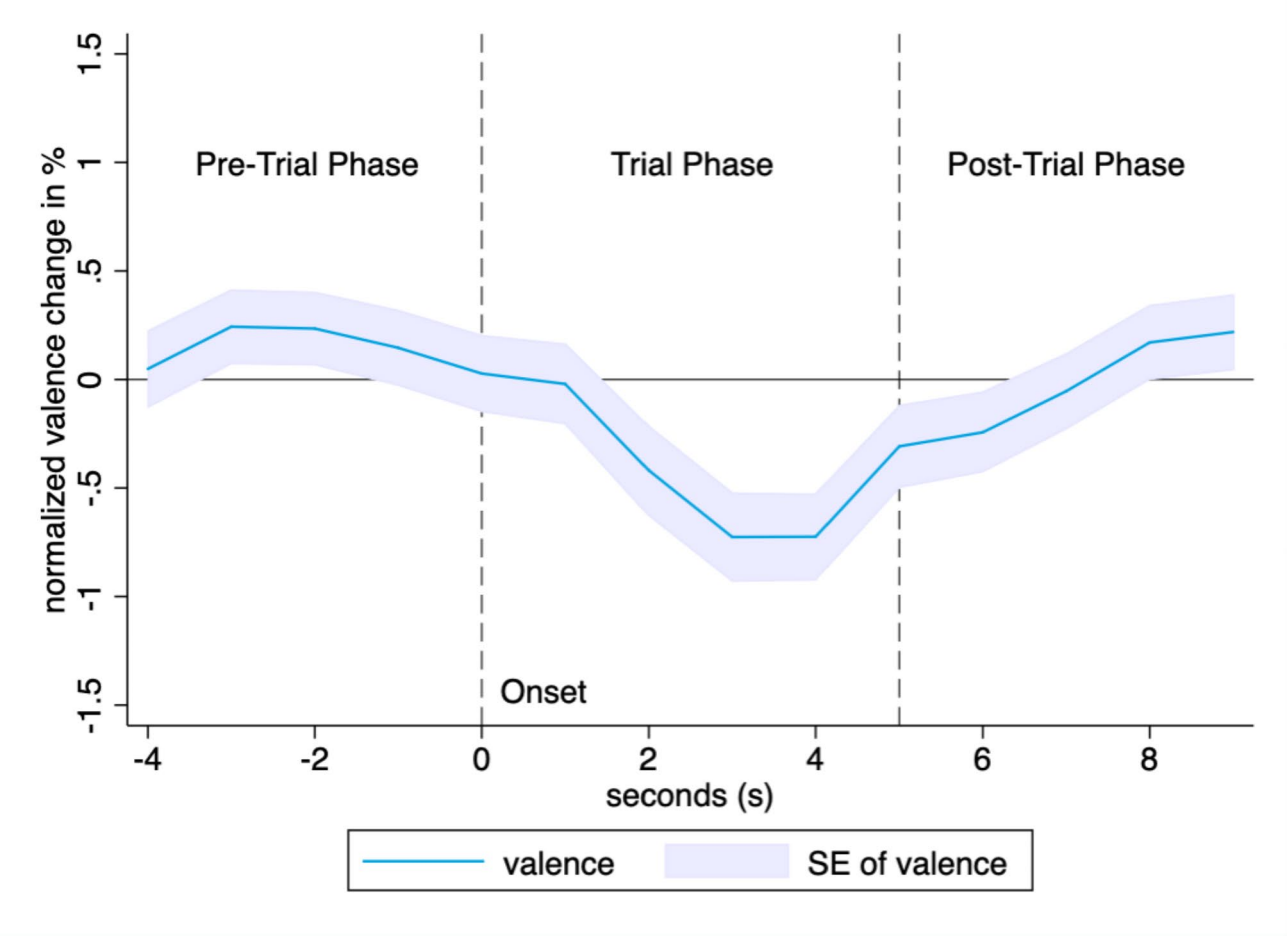
The figure displays the mean valence scores from 5 s before the onset of a trial until 10 s after it. The mean valence scores of participants were computed across all trials performed during the PVT and were plotted in relation to each trial’s onset. To enhance visual clarity, the valence scores were normalized by the pre-trial valence, i.e., -5 to 0 s before trial onset.

The video data in our experiments was sampled at a frequency of 24 frames per second, allowing for a high level of temporal resolution. In order to maintain consistency across trials, we established a uniform temporal frame of reference by selecting the first 5 s after the onset of each trial (i.e., the start of the counter in the PVT) to extract valence and attention scores (as depicted in Fig. 5). The mean values of the extracted scores were then calculated to obtain a single value for each trial, thereby mitigating the influence of intertrial variability on the analysis. This approach has two benefits: Firstly, it enables us to compare valence and attention levels across trials with varying intertrial intervals ranging from 1 to 10 s. Secondly, by focusing on the emotional states that are directly related to and potentially causative of each trial, this approach reduces the potential for spurious associations in the data. In order to facilitate comparisons across trials, the extracted valence and attention values were not normalized.

### Statistical analyses

For the regression models reported in the main “Results” section, we utilized the software STATA 18.0, details of which can be found in the attached code and data. Further, we adopted a panel regression mixed effects approach to account for the temporal structure of the data and conservatively adjusted standard errors at the subject level to appropriately handle repeated measurements. It is noteworthy that our regression models did not include any interactions, and all variables employed are fully reported within the regression tables and no additional covariates were included. Unless specified otherwise, all main results were derived from data aggregated at the trial level. There was no evidence of collinearity among the explanatory variables, and to illustrate the distributional characteristics of the data, we have provided scatter plots of valence and attention plotted against reaction times (collapsed on a subject level) in Supplement S4.

### Study 1 - the Anagram Task

#### Participants

 Participants were recruited randomly from a pool of psychology students at Arizona State University, resulting in a total of 121 participants (65 men, 56 women) between the ages of 18 and 30. Participants provided informed consent and the study protocol was approved by the Institutional Review Board (IRB) at the University of Arizona (#STUDY00005377). All methods were performed in accordance with the relevant guidelines and regulations. Given the innovative nature of our study, which seeks to establish a connection between facial expressions during a sustained attention task and subsequent cognitive performance, the sample size could not be determined based on previously reported effect sizes. To address this issue, we conducted a non-behavioral study to assess the sensitivity of Affectiva in detecting emotions, which is detailed in Supplemental Material S1. After conducting this pilot study, we were confident that a sample size of 121 participants would provide a more reliable means of detecting changes in facial expressions that occur in response to visual stimuli. Participants received course credits as compensation for their participation in both the pilot and main studies. We thoroughly visually inspected all video recordings and excluded nine participants from the final analysis due to *poor video quality.* Specifically, due to poor light conditions in one session, participants’ attention was not consistently decodable from the video footage, resulting in large gaps in the recordings. An additional three participants were excluded from the analysis due to their inability to perform the PVT correctly. Particularly, we excluded subjects who were not clearly visible on at least 40% of the video footage. As a robustness check, we re-analyzed the data set without any exclusions and obtained qualitatively similar results presented in Supplemental Material S3.

#### Behavioral paradigm

 We followed the sequential task paradigm to induce changes in participants’ emotional and attentional states by asking them to perform a prolonged PVT (programmed in E-Prime 3.0), as established in previous studies^16,35^. We randomly assigned them to either the negative or positive feedback group to introduce further exogenous variation in participants’ emotional and attentional states. In the negative feedback group, participants were asked to perform the PVT for 5 min while receiving negative sham feedback, indicating poor task performance and an increased task duration of 20 min. However, feedback was manipulated such that the task always ended after exactly 20 min. The positive feedback group was asked to perform the same vigilance task for 35 min while receiving sham feedback, indicating good task performance and a reduced task duration of 20 min (again, the actual time on the task was set to exactly 20 min for all participants).

We hypothesized that attentional or emotional states of participants would induce behavioral changes in the subsequent tasks. If the treatment successfully created additional variation in attentional and emotional states (holding actual PVT duration constant), we would also observe differences in subsequent task performance between the two treatment groups^1,25^.

To this end, all participants completed a standard Anagram Task after the PVT. Specifically, they were instructed to solve as many anagrams as possible (out of a total of 60) within 360 s. The anagrams were sorted in ascending order of difficulty, ranging from 5 to 7 letters, and excluded proper names and homonyms. Participants completed three practice trials of short anagrams and were not permitted to ask any questions once the practice trials were completed. There was no direct treatment effect across both groups (*p* > .1), as participants in the positive treatment group solved on average 19.47 (*N* = 51; SD = 8.12) compared to the negative group with an average of 19.29 (*N* = 58; SD = 7.22) solved anagrams. The task was fully computerized and programmed in E-Prime 3.0.

### Study 2 - the Ultimatum Game

#### Participants

 We collected data from 96 participants (41 men, 54 women, and 1 non-binary) with a median age of 18.8 years (SD = 1.40) recruited from a pool of psychology students at Arizona State University. Due to technical issues with the cameras, we were not able to collect any video footage from 24 participants and this data was therefore not analyzable. Participants provided informed consent and the study protocol was approved by the Institutional Review Board (IRB) at the University of Arizona (#STUDY00005377). All methods were performed in accordance with the relevant guidelines and regulations.

We excluded four participants due to insufficient analyzability of their video footage, and an additional two participants were excluded from the analysis due to their inability to perform the PVT correctly. Therefore, the final analysis included 90 participants. Participants earned an average of $8.48 (SD = 3.22) in the experiment, plus an additional show-up payment of $4.

#### Behavioral paradigm

 In Study 2, we utilized the same experimental design and paradigm as in Study 1 to explore the effect of valence and attention on far-transfer task performance. The same treatments were applied to manipulate valence and attention between experimental groups, as detailed in the “Methods” section of Study 1. However, for Study 2, we employed an Ultimatum Game (UG) as a far transfer task^62^. Participants were randomly assigned the role of sender or responder and played the UG for two rounds in a fully computerized and anonymous setting using z-Tree (version 5.1.15)^63^. At the start of each round, participants were paired with a stranger while holding the roles of sender or responder constant. Senders were given $10 and required to propose an offer to split the money with the responder in whole dollar amounts. The responder could either accept or reject the offer and if accepted, the money was split as proposed. If rejected, neither of them received any money in that round. Further, we introduced a unique feature in the design that deviated from the standard UG to increase the variability of offers. Specifically, based on previous literature, we hypothesized that self-control lapses would be more frequent when participants reacted hotly to a low offer, rather than when they coldly deliberated on hypothetical minimal acceptable offers^34^. Therefore, in each round, responders were presented with one real and in addition, one low fake offer, with no information provided on which offer was real. All participants were ex-ante fully informed about this procedure. This allowed us to tap into the “hot” state response when given a low offer and allowed us to concentrate on rejection rates, which are expected to be more influenced by affective “hot” states of participants^34,43^. Further, we limited our analysis to the responder’s behavior, as previous research suggests that altruistic punishment (punishment of unfair behavior) is closely linked to emotional reactions to perceived unfairness^34,43^.

## Electronic supplementary material

Below is the link to the electronic supplementary material.

Supplementary Material 1

## Data Availability

Supplementary information is available for this paper. Behavioral data and measurements derived from video footage (valence and attention), as well as the reported statistical analyses, are included in the supplementary information files of this article. Raw video files cannot be included due to participant confidentiality. Correspondence and requests for materials should be addressed to gene.brewer@asu.edu.
