# Supplementary material for: Decoding the influence of emotional and attentional states on self-control using facial analysis
Source: Sci Rep. 2024 Oct 26;14:25465. doi: 10.1038/s41598-024-73729-6 (PMC11513103; doi:10.1038/s41598-024-73729-6)
Supplement: Supplementary file 1 — Supplementary Material 1 [file 41598_2024_73729_MOESM1_ESM.docx]

Supplementary Information

Decoding the Influence of Emotional and Attentional States on Self-Control Using Facial Analysis

Gökhan Aydogan^1,*^, Janek Kretschmer^2,*^, Gene Brewer^3,✝^,

Samuel M. McClure^3^

### S1. Emotions during a movie scene from “Something about Mary”

**
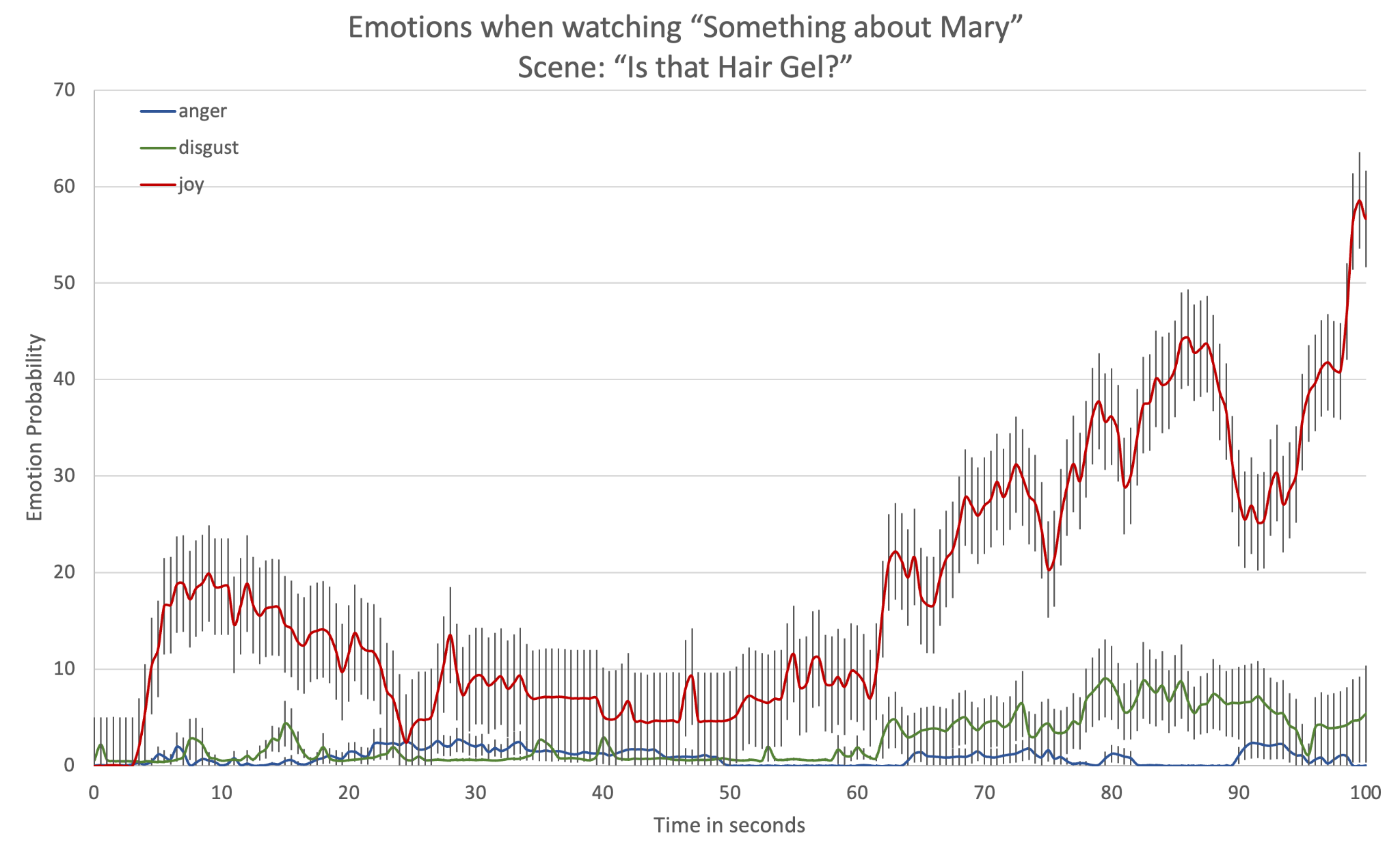
Fig S1.** 45 participants’ facial reactions were recorded and analyzed while they were watching a scene from the movie “Something about Mary”.[^1^](https://paperpile.com/c/PwiGRX/cVza) The graph shows for each emotion (anger, disgust, joy) its mean and standard error at each time point during the movie clip (MATLAB 24.1).

To test the effectiveness of Affectiva and whether it fits our purposes to detect emotional reactions from faces, we invited 45 participants to watch a scene from “Something about Mary”. The participants’ facial expressions were recorded while they were watching the 100-second-long movie clip. Figure S1 shows the emotions anger, disgust, and joy over the whole course of the clip. While virtually no anger was detected, Affectiva showed joy and disgust, which peaked at specific time points during the movie clip.

### S2. Psychomotor vigilance task with positive and negative performance feedback

In this study, we asked participants to perform the psychomotor vigilance task (PVT) and randomly assigned them to two treatment groups, in which we manipulated participants’ motivation to perform this task. In both treatment groups, participants were instructed to monitor a computerized stopwatch, which begins counting (in milliseconds) at random intervals on the screen. Participants were told that they needed to press the spacebar as soon as the clock on the screen started counting. The PVT is a simple reaction time task and places minimal demands on the cognitive system.

However, depending on the treatment group, we additionally manipulated participants’ motivation by providing them either with positive or negative feedback after each trial during the task.

*The positive feedback treatment*:

In the treatment group with positive feedback (or gain treatment), participants were told that they had to perform the PVT for *35 minutes* but were able to reduce their time if they maintained a certain performance level. Specifically, participants were able to reduce their time by 10 seconds for each successful trial. Thus, due to the low task demands, participants received after most trials positive feedback. Additionally, they received *no* feedback regarding the actual time that passed during the task and were not allowed to use cell phones or watches during the experiment. To hold the actual time on the task constant across all participants (and treatment groups), the feedback was manipulated in a way that the task always finished exactly after *20 minutes*.

*The negative feedback treatment*:

In the negative feedback group (or loss treatment), participants were asked to perform the PVT for 5 minutes but were told that they needed to maintain a certain performance level (i.e., reaction time faster than 275ms). Otherwise, their time on the task would increase by 10 seconds for each lost trial. However, again to hold the actual time on this task constant, the experiment finished after 20 minutes, irrespective of the actual performance. Again, participants were led to believe that their (bad) performance increased the time to 20 minutes.

### S3. Supplementary Analyses

To confirm that the exclusion of participants with poor recording quality had no impact on the results reported under Study 1, we re-analyzed the data with all available subjects. Table S1 shows the results with all available data. However, please note that due to listwise deletion, there are still 4 subjects dropped from the analysis.

**Table S1.**

Predicting anagram performance

| Predictor | Dependent Variable:  Correctly solved anagram puzzles | |
| --- | --- | --- |
|  | Model (1) | Model (2) |
| Treatment condition  (1=pos. or 0=neg. feedback) | .608  (1.448) | .413  (1.449) |
| **Attention during PVT** |  | **.106****  **(.038)** |
| Valence during PVT |  | .013  (.022) |
| Constant | 19.257**  (1.002) | 9.383*  (3.423) |
| Log Likelihood | -34,615.271 | -34,554.162 |

Note: The table reports unstandardized coefficients of mixed regression, with robust standard errors in parentheses (*N* = 117). **p* < .05. ***p* < .01 (Stata 18.0).

### S4. Relationship of Attention and Valence with RTs

To examine the relationship between reaction times (RTs) with attention or valence in the experiments, we regressed RTs on either valence or attention for each condition (positive or negative) separately (see Figure S2). Our results indicate a negative relationship between RTs and attention (or valence), demonstrating that participants with much larger reaction times (indicating lower task engagement) showed lower attention and valence levels.


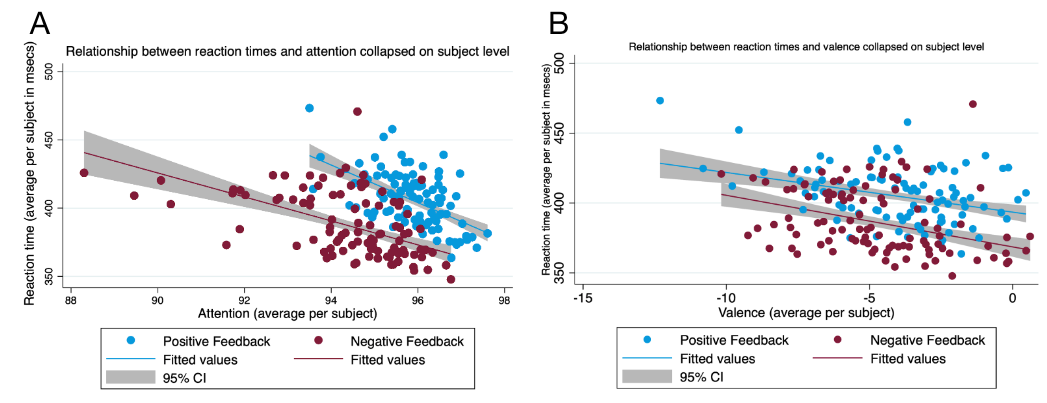


### Fig S2. Depicted are attention (Panel A) and valence (Panel B) levels plotted against reaction times (averaged over all trials and collapsed on subject level). Respectively, mixed effects regressions with clustered standard errors on the subject level indicate that higher valence (*β_valence_* = -10.3, *p* < .001) or attention (*β_Attention_* = -3.3, *p* < .001) are significantly associated with lower reaction times. Additionally, a main effect of condition (p < .05) on reaction times was observed. As expected, reaction times were longer in the positive feedback condition, which was by design much easier. Conversely, the short reaction times in the negative feedback group suggest that participants exhibited at least the same engagement (or higher) as in the positive feedback group, supporting the effectiveness of our deception strategy.

###

### S5. Experimental Instructions

***Ultimatum Game: General Instructions***

*This experiment is a simple bargaining game that you will play with the other participants at today’s session. Each time you play, you will be paired with a partner in a one-shot decision. In other words, you will bargain with each partner only once.*

*The game will consist of two rounds. At the beginning of round 1, you will be randomly assigned a role of either “sender” or “responder”. This will be your role for the entirety of the game. In other words, you will not get the opportunity to play both roles.*

*At the start of each round, senders and responders will be randomly paired up to bargain over an amount of $10. The sender will make his or her decision first by offering some part of the amount to the responder.*

*If you are a sender, you will write an offer in the appropriate box on your screen. This number must be between $0 and $10 (inclusive). This offer will be sent to the responder. The responder will then have to choose either to accept or decline the offer by clicking on the appropriate button. If the offer is accepted, the responder gets the offered amount of dollars and the sender gets $(10 – offer). If the offer is declined, the sender and the responder both get nothing (i.e., $0).*

*For example, suppose Al is a sender, and Vernon is a responder. Al offers Vernon $7. If Vernon accepts, Al gets $3, and Vernon gets $7. If Vernon chooses “declines,” both Al and Vernon get $0. After all participants have made their decisions, we will continue with the next part.*

***Ultimatum Game: Sender.***

*You are in the role of the “sender”.*

***Your decision.***

*You are randomly matched to another participant. What split of $10 do you offer the “responder”? If the responder accepts your offer, you will keep $10 - the “offer”.*

*The other person decides whether to accept or reject the “offer” you send to him/her. The other person is also informed about his or her earnings and your earnings in case the person accepts or rejects the offer.*

***Ultimatum Game: Responder.***

*You are in the role of the “responder”.*

***Your decision.***

*You are randomly matched to another participant. The other person is asked to $10 between you and himself/herself. We ask you to decide whether to accept or reject the “offer” the other person sent to you.*

***Accept.***

*In case you accept the amount the other person sent, your earnings are the amount the other person sent to you. In that case, the earnings of the other person are the remainder of the $10, so $10 - the “offer”.*

***Reject.***

*If you reject the offer, you both earn nothing.*

***Additional information for responders.***

*If you are a responder, you will see in total 4 offers. Two of your offers will be fictional and two offers will be real. We would like you to treat each offer as if it is real. Note that, for all you know, each offer could be real, so your best strategy is to treat every offer as if it was going to be implemented.*

***Hypothetical minimal acceptable offer.***

*Your role was “responder”. Hypothetically speaking, what would have been the offer that you would have accepted from the sender? That is, what is the smallest amount you would have accepted if it was offered by the sender?*

***Additional question.***

*In your opinion, what do you believe was the average amount that was sent in this session? If your estimate is correct (give or take 0.5), you will earn an additional $2.*

**Instructions Anagram Task and Psychomotor Vigilance Task**

Instructions for those tasks were presented on the screen.

# References

1. [Farrelly, B. *et al.* *There’s Something about Mary*. (Skífan, 1998).](http://paperpile.com/b/PwiGRX/cVza)
